# Non-Debye Behavior of the Néel and Brown Relaxation in Interacting Magnetic Nanoparticle Ensembles

**DOI:** 10.3390/ma17163957

**Published:** 2024-08-09

**Authors:** Cristian E. Botez, Jeffrey Knoop

**Affiliations:** Department of Physics and Astronomy, University of Texas at San Antonio, 1 UTSA Circle, San Antonio, TX 78249, USA; jeff.knoop@my.utsa.edu

**Keywords:** ferrofluids, ac-susceptibility, superspin relaxation, nanoparticle ensembles

## Abstract

We used ac-susceptibility measurements to study the superspin relaxation in Fe_3_O_4_/Isopar M nanomagnetic fluids of different concentrations. Temperature-resolved data collected at different frequencies, χ″ vs. T|_f_, reveal magnetic events both below and above the freezing point of the carrier fluid (T_F_ = 197 K): χ″ shows peaks at temperatures T_p1_ and T_p2_ around 75 K and 225 K, respectively. Below T_F_, the Néel mechanism is entirely responsible for the superspin relaxation (as the carrier fluid is frozen), and we found that the temperature dependence of the relaxation time, τ_N_(T_p1_), is well described by the Dorman–Bessais–Fiorani (DBF) model: $\tau_{N}\left(T\right)=\tau_{r}\exp\left(\frac{E_{B}+E_{ad}}{k_{B}\;T}\right)$. Above T_F_, both the internal (Néel) and the Brownian superspin relaxation mechanisms are active. Yet, we found evidence that the effective relaxation times, τ_eff_, corresponding to the T_p2_ peaks observed in the denser samples *do not* follow the typical Debye behavior described by the Rosensweig formula $\frac{1}{\tau_{eff}}=\frac{1}{\tau_{N}}+\frac{1}{\tau_{B}}$. First, τ_eff_ is 5 × 10^−5^ s at 225 K, almost three orders of magnitude more that its Néel counterpart, τ_N_~8 × 10^−8^ s, estimated by extrapolating the above-mentioned DBF analysis. Thus, $\frac{1}{\tau_{N}}\gg \frac{1}{\tau_{eff}}$, which is clearly not consistent with the Rosensweig formula. Second, the observed temperature dependence of the effective relaxation time, τ_eff_(T_p2_), is excellently described by $\tau_{B}^{-1}\left(T\right)=\frac{T}{\gamma_{0}}\exp\left[-\frac{E^{\prime }}{k_{B}\left(T-{T_{0}}^{\prime }\right)}\right]$, a model solely based on the hydrodynamic Brown relaxation, $\tau_{B}(T)=\frac{3\eta \left(T\right)V_{H}}{k_{B}T}$, combined with an activation law for the temperature variation of the viscosity, $\eta \left(T\right)=\eta_{0}\exp\left[E^{\prime }/k_{B}(T-{T_{0}}^{\prime }\right]$. The best fit yields $\gamma_{0}=\frac{3\eta V_{H}}{k_{B}}$ = 1.6 × 10^−5^ s·K, E′/k_B_ = 312 K, and T_0_′ = 178 K. Finally, the higher temperature T_p2_ peaks vanish in the more diluted samples (δ ≤ 0.02). This indicates that the formation of larger hydrodynamic particles via aggregation, which is responsible for the observed Brownian relaxation in dense samples, is inhibited by dilution. Our findings, corroborating previous results from Monte Carlo calculations, are important because they might lead to new strategies to synthesize functional magnetic ferrofluids for biomedical applications.

## 1. Introduction

The applications of magnetic nanoparticles in biomedicine have grown exponentially over the past two decades, particularly in fields of critical importance, such as medical imaging [1,2,3], drug delivery [4,5,6], biosensing [7,8,9], and tissue engineering [10,11]. Yet, the application with the most transformative potential remains magnetic hyperthermia [12,13,14,15], a procedure that can kill malignant tumors without the side effects of chemo and radiation therapy. Indeed, it is well established that cancer cells die at temperatures just a few degrees below their normal cell counterparts, so bringing the temperature within the 42–45 °C range selectively kills malignant tumors without affecting the adjacent healthy tissue. The idea of using nanomagnets to achieve such heating dates back to 1957, when Gilchrist demonstrated for the first time the ability to heat lymph node cancer using magnetic nanoparticles in an alternating magnetic field [16]. Unfortunately, practical issues related to delivering and attaching the nanoparticles to the tumor, as well as to ensuring uniform heating, have limited the applicability of the method for several years, but technological advances led to a revived interest in magnetic nanoparticle hyperthermia throughout the 1990s and early 2000s [17,18]. Ferromagnetic nanoparticles were initially used, but it was quickly noticed that these systems could not generate enough heat within the limits that need to be imposed on the amplitude and frequency of the driving magnetic field (H·f < 10^6^ Oe·Hz). The reason is that the heat-dissipation power of ferro or ferrimagnetic nanoparticles, measured based on their specific absorption rates (SARs), is proportional with the area of the hysteresis loops. Even if strong Co-based materials are used (which adds biocompatibility issues), the SARs are still two orders of magnitude below what would be needed for clinical trials. To address this, the use of superparamagnetic nanoparticles [19] (SPNs) was proposed. Heat generation in SPNs is not hysteretic, but depends on the collective dynamics of the nanoparticles’ giant magnetic moments, the superspins. At each temperature, T, the superspins flip via thermal activation (within the nanoparticle) over a so-called energy barrier to magnetization reversal, E_B_ [20]. The phenomenon is called Néel relaxation, and the relaxation time, τ_N_—the time it takes the superspin to complete one full rotation by flipping twice along an easy magnetization axis—depends on the inverse temperature 1/T, through an Arrhenius activation law. For an ideal system of isolated (non-interacting) and monodisperse SPNs, the Néel relaxation is given by $\tau_{N}(T)=\tau_{0}\exp\left(\frac{E_{B}}{k_{B}T}\right)$ [21], whereas for weak and medium-strength interparticle interactions it becomes the following:

(1) $$\tau_{N}\left(T\right)=\tau_{r}\exp\left(\frac{E_{B}+E_{ad}}{k_{B}\;T}\right)$$

Equation (1) was first proposed by Dormann, Bessais, and Fiorani (DBF) [22]. It introduces an energy barrier to superspin reversal that includes contributions from (1) the nanoparticles’ material and average size, through E_B_ = KV (K is the magnetic anisotropy and V is the average nanoparticle volume), and (2) the interparticle magnetic dipolar interactions, through E_ad_. K depends on the nanoparticles’ material, as well as on their average size due to an additional surface anisotropy component. The SARs associated with the Néel mechanism always increase with the value of the overall energy barrier. In other words, the harder it is to flip the superspin within a nanoparticle, the more heat the SPN ensemble is able to generate. Consequently, research efforts have been aimed at designing and synthesizing SPN ensembles with increased energy barriers to magnetization reversal [20]. At the same time, it was noticed that whenever the nanoparticles are delivered through intravenous injection and magnetically driven to the tumor, a second type of superspin relaxation—the Brown relaxation—can be active in addition to the Néel mechanism. Indeed, since the nanoparticles are not fixed in a solid matrix, but immersed in a fluid, the superspin can also flip under the action of the driving alternating magnetic field through the physical rotation on the nanoparticles in the fluid. The Brown relaxation time is given by the following:

(2) $$\tau_{B}\left(T\right)=\frac{3\eta \left(T\right)V_{H}}{k_{B}T}$$

where η is the fluid’s temperature-dependent viscosity, V_H_ is the average hydrodynamic volume of the nanoparticles, and k_B_ = 1.3806 × 10^−23^ J/K is the Boltzmann constant.

When both the Néel and the Brown relaxation processes are active in an SPN ensemble immersed in a carrier fluid, a Debye-type model [23] is often used to describe the combined contribution of the two mechanisms. In this case, an effective relaxation time τ_eff_ that depends on τ_N_ and τ_B_ was introduced by Rosensweig [24]:

(3) $$\frac{1}{\tau_{eff}}=\frac{1}{\tau_{N}}+\frac{1}{\tau_{B}}$$

Equation (3) has been extensively used to study the superspin relaxation in ferrofluids, separate the individual contributions of the Néel and the Brown relaxation, and determine which one is dominant [25,26]. In recent years, however, several studies [27,28] have pointed out that the Rosensweig formalism was developed to describe the relaxation of non-interacting nanoparticles in the absence of any magnetic field, and, consequently, its ability to accurately describe the collective dynamic behavior of the superspins in dense ferrofluids under the action of alternating magnetic fields needs further examination. Moreover, τ_N_ and τ_B_ have a markedly different dependence on the temperature and the SPNs’ average size (e.g., τ_N_ depends exponentially on the magnetic volume, whereas τ_B_ depends linearly on the hydrodynamic volume). Therefore, their interplay as described by Equation (3) might change significantly upon the change in the SPN structural characteristics and external stimuli. Accordingly, recent computational investigations have predicted that dipolar interactions among the nanoparticles in the ensemble lead to deviations from the Debye behavior [29], and ac-hysteresis measurements showed that such interactions play an important role in magnetic losses [30]. Other experiments have revealed evidence that the Brownian relaxation occurs under conditions where only its Néel counterpart was expected [31].

Here, we report results on the superspin relaxation in ferrofluids of different concentrations synthesized by dispersing 15 nm-average-diameter Fe_3_O_4_ nanoparticles in Isopar M. We measured the temperature (T) dependence of the out-of-phase ac-susceptibility (χ″) upon heating from 3 K to 300 K at four different frequencies (f). These χ″ vs. T|_f_ curves exhibit peaks that shift toward higher temperatures upon the increase in the measurement frequency, a feature typically used to uncover information on the magnetization dynamics of an SPN ensemble [32]. In the dense samples, our data show two such peaks, T_p1_ and T_p2_, at temperatures around 75 K and 225 K, respectively. Interestingly, the higher-temperature peak vanishes in samples of low concentrations, below 0.04 mg/mL. The lower-temperature peak T_p1_ is associated with the Néel relaxation, as the carrier fluid freezes at T_F_ = 197 K, so the Brown mechanism cannot be active below this temperature. We fit the observed temperature dependence of the relaxation time, τ_N_(T_p1_), using the DBF model (Equation (1)). This allowed us to determine the overall barrier to superspin reversal associated with the Neel relaxation, E_B_ + E_ad_. Using data collected on the most diluted sample, we found (E_B_ + E_ad_)/k_B_ = 900 K. Our most important findings come from the analysis of the high-temperature peaks T_p2_ in dense samples (concentrations above 0.04 mg/mL). In this case, both the Néel and the Brown relaxation could be active, but we found clear evidence that the measured effective relaxation times, τ_eff_, *do not* follow the typical Debye behavior described by the Rosensweig formula. Specifically, using data collected on the densest sample we found that (1) τ_eff_ is 5 × 10^−5^ s at 225 K, i.e., three orders of magnitude more that its Néel counterpart, τ_N_~8 × 10^−8^ s. Thus $\frac{1}{\tau_{N}}\gg \frac{1}{\tau_{eff}}$, which is not consistent with the Rosensweig formula, (2) the observed temperature dependence of the effective relaxation time, τ_eff_(T_p2_), is excellently described by $\tau_{B}^{-1}\left(T\right)=\frac{T}{\gamma_{0}}\exp\left[-\frac{E^{\prime }}{k_{B}\left(T-{T_{0}}^{\prime }\right)}\right]$, a model solely based on the hydrodynamic Brown relaxation combined with an activation law for the temperature variation of the viscosity, so the Néel relaxation appears to *not* play a role at temperatures around 225 K, and (3) the T_p2_ peaks vanish in the highly diluted samples, which indicates that the formation of large hydrodynamic particles via aggregation (responsible for the observed Brownian relaxation in denser samples) is inhibited by dilution. These results are important because they provide further experimental evidence to support the hypothesis that non-Debye relaxation occurs in interacting ferrofluids. Confirming this behavior in more complex magnetic nanoparticles (e.g., ZnFe_2_O_4_ and MnFe_2_O_4_) that are being tested for cancer research [33] might lead to new approaches to design ferrofluids for magnetic hyperthermia therapy.

## 2. Materials and Methods

All ferrofluids used in this study were prepared using 15 nm-average-diameter Fe_3_O_4_ nanoparticles coated with oleic acid and dispersed in Isopar M. We synthesized five samples of different concentrations. The first, as-prepared sample (δ = 1), contains 10 mg of nanoparticles and 5 mL of carrier fluid. The other four samples, δ = 0.2, δ = 0.02, δ = 0.013, and δ = 0.01, were made via the progressive dilution of the as-prepared sample by simply increasing the Isopar M volume 5, 50, 75, and 100 times, respectively. The chemical and structural quality of the nanoparticle ensembles was assessed via (1) powder x-ray diffraction (XRD) using synchrotron x-rays of wavelength λ = 0.711 Å at the National Synchrotron Light Source (Brookhaven National Laboratory) and (2) Transmission Electron Microscopy (TEM) using a Hitachi H-9500 microscope (Hitachi, Schaumburg, IL, USA) operating at 300 kV. Ac-susceptibility measurements were carried out on ferrofluid samples placed in a variable-frequency (10 Hz −10,000 Hz) alternating magnetic field of amplitude 3 Oe generated by a Quantum Design^®^ Physical Property Measurement System (Quantum Design, San Diego, CA, USA). Data were collected upon heating in 5 K steps from 5 K to 300 K. At all temperatures, the in-phase (χ′) and out-of-phase (χ″) components of the susceptibility were recorded at four different values of the driving magnetic field frequency (f), 300 Hz, 1000 Hz, 3000 Hz, and 10,000 Hz. 

## 3. Results and Discussion

**Figure 1:**
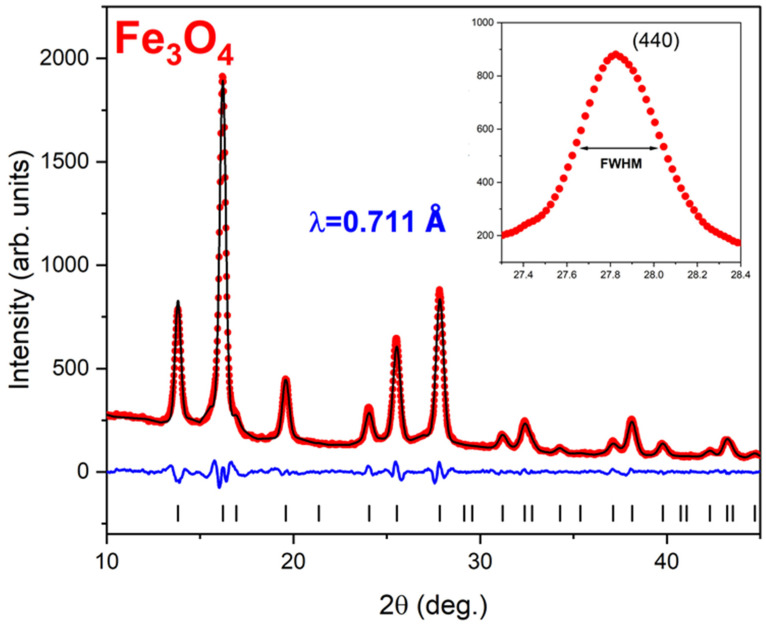
X-ray powder diffraction data, I_obs_ vs. 2θ, measured on the Fe_3_O_4_ nanoparticle ensemble using synchrotron x-rays of wavelength λ = 0.711 Å (red symbols). The solid line shows the angular dependence of the calculated intensity via a Le Bail (full profile) fit. The vertical bars show the angular positions of the Bragg reflections, and the blue lower trace is the difference curve I_calc_–I_obs_. The inset shows the (440) Bragg peak, for which the full width at half maximum (FWHM) was used to calculate the average diameter, <D>, of the nanoparticles.

Figure 1 shows the x-ray diffraction (XRD) pattern, I_obs_ vs. 2θ, collected using synchrotron radiation of wavelength λ = 0.711 Å from an Fe_3_O_4_ powder obtained from the dense δ = 1 sample by evaporating the carrier fluid (red symbols). The solid black curve is the calculated XRD profile, I_calc_ vs. 2θ, corresponding to the best full-profile (Le Bail) fit [34] to the data.

**Figure 2:**
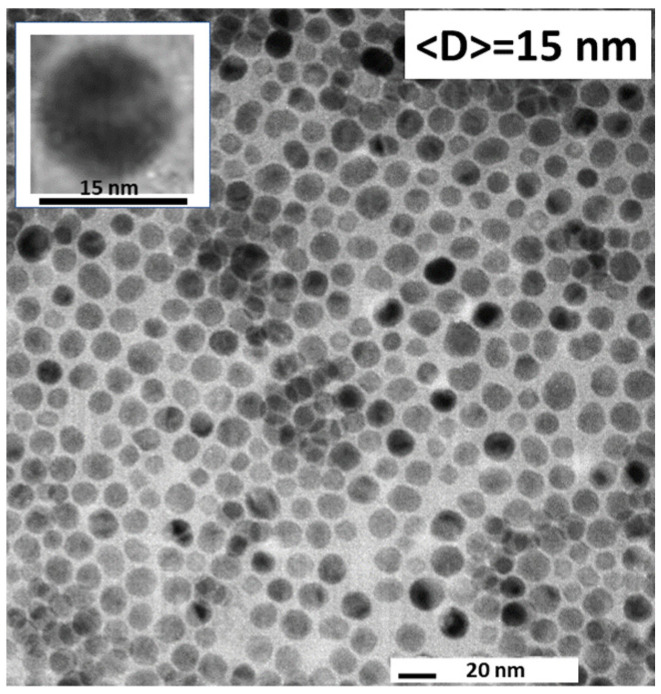
Transmission Electron Microscopy (TEM) data measured on Fe_3_O_4_ nanoparticles yields an average diameter <D> = 15 nm. The inset shows the image of a single spherical particle from the ensemble.

The fit, carried out using the program FULLPROF [35], converges to low residuals upon the variation of five independent parameters related to the lattice constant and the peak profiles. The latter were modeled using a pseudo Voigt function. The vertical lines show the angular, 2θ, positions of the Bragg reflections, and the lower blue curve shows the difference between the calculated and the observed intensities, I_calc_–I_obs_. The fit confirms the chemical and structural purity of the nanoparticles through the presence of only one phase: cubic Fe_3_O_4_ S.G. Fd3m, a = 8.360 Å. In addition, this analysis allowed us to determine the average diameter of the nanoparticles, <D>. The inset shows, in more detail, the peak corresponding to the (440) reflection, for which the full width at half maximum is FWHM = 0.25 deg = 0.004 rad. Using Scherrer’s formula [36] $<D>=\frac{0.9\cdot \lambda }{FWHM\cdot cos\frac{2\theta }{2}}$, we found <D > = 15 nm. Figure 2 presents a TEM image recorded on an Fe_3_O_4_ nanoparticle ensemble from the δ = 1 magnetic fluid. The inset shows a single nanoparticle of a nearly perfect spherical shape demonstrating the quality of the sample’s morphology.

In addition, the TEM data confirm the value of the average diameter obtained from the XRD data. It is also important to indicate that the results of the XRD and TEM analyses above carry over to the other samples used our study (δ = 0.2, δ = 0.02, δ = 0.013, and δ = 0.01). All ferrofluids were synthesizes via the progressive dilution of the as-prepared one, so they contain Fe_3_O_4_ nanoparticle ensembles with the same chemical, structural, and morphological characteristics.

**Figure 3:**
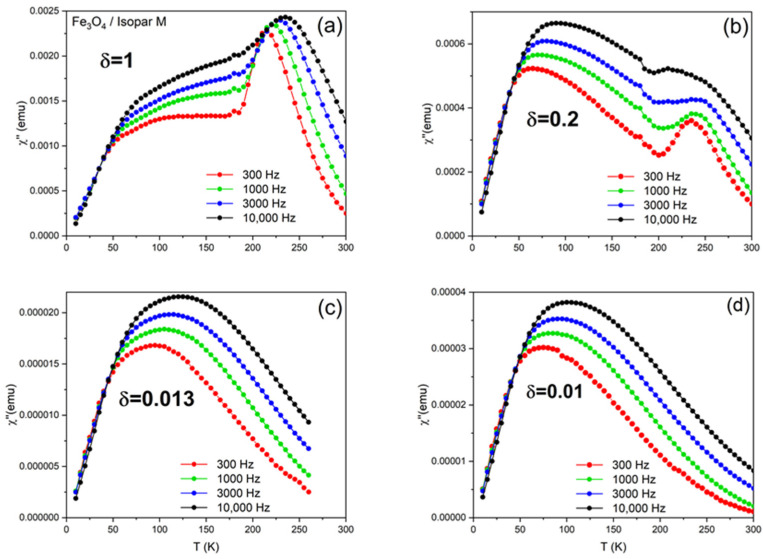
Temperature-resolved ac-susceptibility data measured at different frequencies spanning a three-order-of-magnitude range, 300 Hz (red), 1000 Hz (green), 3000 Hz (blue), and 10,000 Hz (black) on Fe_3_O_4_/Isopar M ferrofluids of different concentrations: (**a**) δ = 1, the as-prepared sample made of 10 mg of nanoparticles dispersed in 5 mL of carrier fluid, (**b**) δ = 0.2, (**c**) δ = 0.013, and (**d**) δ = 0.01.

The observed temperature dependence of the out-of-phase susceptibility measured at different frequencies of the driving magnetic field, χ″ vs. T|_f_, is shown in Figure 3 for (a) δ = 1, the as-prepared sample, (b) δ = 0.2, (c) δ = 0.013, and (d) δ = 0.01. Ac-susceptibility data are critical for the study of the magnetization dynamics and have been used extensively in investigations of the superspin relaxation in systems of SPNs [37]. χ″ vs. T|_f_, in particular, shows a peak whenever the observation time, which is controlled by the measurement frequency via τ_obs_ = 1/2πf, is equal to the superspin relaxation time, τ, which is strongly influenced by the temperature. Interestingly, χ″ vs. T|_f_ exhibits a peak when τ_obs_= τ regardless of how complex the superspin relaxation process is, including the case when the Néel and Brown mechanisms act concomitantly [38]. The main feature of the data in Figure 3 is that the χ″ vs. T|_f_ curves show two peaks for the dense ferrofluids δ = 1 and δ = 0.2. At any measurement frequency, the lower-temperature peaks are around 75 K, whereas their higher-temperature counterparts are around 225 K. The freezing point of the carrier fluid, Isopar M, is T_F_ = 197 K, so the low-temperature peaks can only be due to the internal Néel relaxation as the physical rotation of the nanoparticles is inhibited by the frozen fluid. At 225 K, however, both the Néel and the Brown mechanisms can be simultaneously active, case in which the overall “effective” superspin relaxation time, τ_eff_, would include contributions from both mechanisms. Another remarkable feature of the ac-susceptibility data is that the high-temperature peaks completely vanish in the highly diluted samples, δ = 0.013 and δ = 0.01, indicating that the processes responsible for the collective superspin relaxation observed around 225 K in the dense nanoparticle ensembles are fully inhibited in the absence of strong interparticle interactions. 

Finally, we note that the temperatures of both sets of peaks, T_p1_ (around 75 K) and T_p2_ (around 225 K), shift upwards upon the increase in the alternating magnetic field frequency from f = 300 Hz (red symbols) to 1000 Hz (green symbols), 3000 Hz (blue symbols), and 10,000 Hz (black symbols). This is important, because it allows us to determine the temperature dependence of the relaxation time, $\tau \left(T\right),$ and compare it with predictions made by phenomenological models in order to get more insight into the microscopic details of the superspin dynamics, as described below.

**Figure 4:**
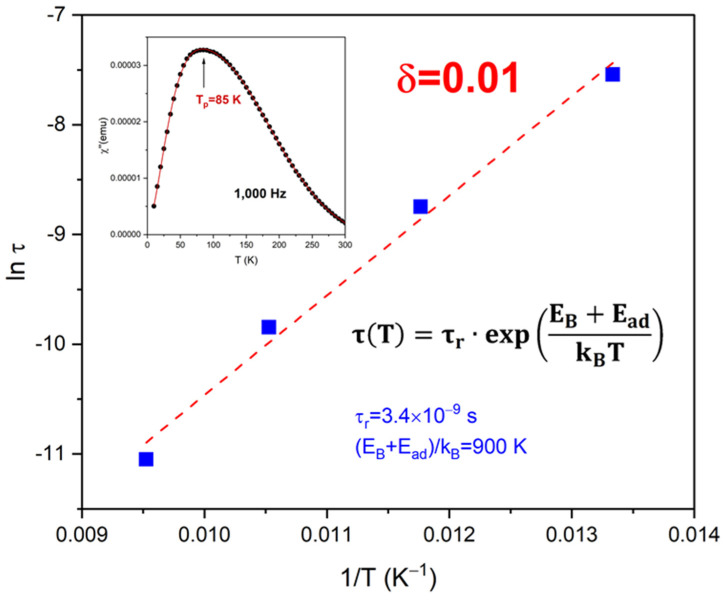
Relaxation time, τ = 1/2πf, of the highly-diluted ferrofluid, δ = 0.01, as a function of the inverse temperature 1/T. The temperature corresponding to each frequency was determined from the peak of the respective χ″ vs. T|_f_ curve, as shown in the inset (black symbols). The solid blue squares show the data, whereas the red dashed line is the best fit to an activation type law $\tau \left(T\right)=\tau_{r}\exp\left(\frac{E_{B}+E_{ad}}{k_{B}T}\right)$.

The solid symbols (blue squares) in Figure 4 show the superspin relaxation time as a function of temperature in the most diluted sample (δ = 0.01) obtained from the ac-susceptibility data in Figure 3d. At each frequency or, equivalently, observation time, the temperature of the peak was determined form a polynomial fit as shown in the inset to Figure 4, where the solid black circles are the χ″ vs. T|_1000 Hz_ data and the solid red line is the best fit. As indicated above, the ac-susceptibility data from the highly diluted samples, such as δ = 0.01, only exhibit the low-temperature peak T_p1_, and only the Néel relaxation mechanism is active at these temperatures because the carrier fluid is frozen. Therefore, we analyzed the τ_N_(T_p1_) data using the DBF model. The dashed line shows the best fit of Equation (1) to the observed temperature dependence of the Néel relaxation time. Allowed to vary in the fit were the pre-factor τ_r_ and the total energy barrier to magnetization reversal E_B_ + E_ad_. The fit converges to low residuals and yields τ_r_ = 3.4 × 10^−9^ s and (E_B_ + E_ad_)/k_B_ = 900 K. These results are significant in at least two respects. First, the quality of the fit to the DBF phenomenological model confirms the low-to-medium level of the interparticle interaction strength in the ferrofluids used in this study, as, typically, strong interactions can only be modeled using a Vogel–Fulcher type law [39]; otherwise, fits to the τ_N_(T) data yield unphysically short pre-factor values [40]. Second, knowledge of the barrier to the magnetization reversal value in these nanoparticle ensembles allows us to estimate their Néel relaxation time (via extrapolation) throughout the entire temperature range used in this study.

**Figure 5:**
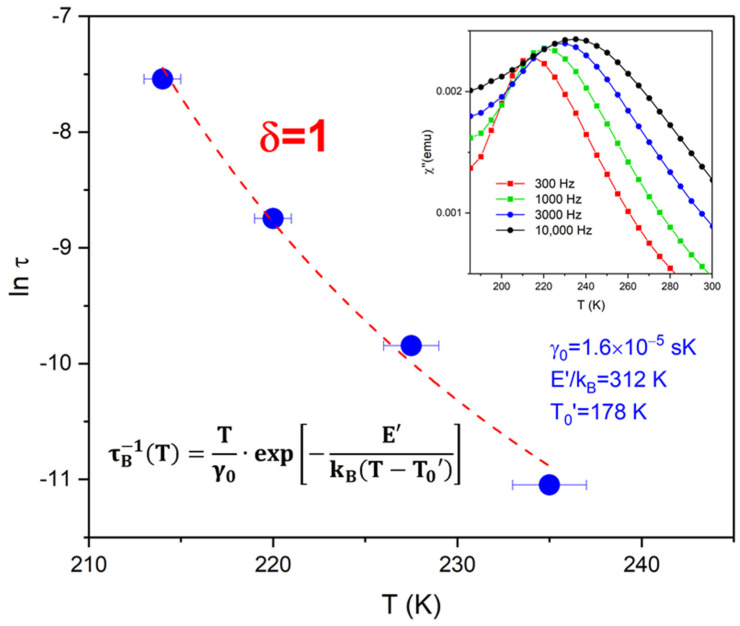
The filled circles show the temperature dependence of the effective relaxation time, τ_eff_ vs. T, determined from the shift of the χ″ vs. T|_f_ peak with the measurement frequency f (inset). The dashed curve is the best fit of an equation based on the hydrodynamic Brown relaxation model: $\tau_{B}^{-1}\left(T\right)=\frac{T}{\gamma_{0}}\exp\left[-\frac{E^{\prime }}{k_{B}\left(T-{T_{0}}^{\prime }\right)}\right]$.

The data and analysis related to the magnetization dynamics of the dense δ = 1 ferrofluid are presented in Figure 5. The solid symbols (blue circles) show the temperature dependence of the relaxation time obtained from the T_p2_ peaks of the χ″ vs. T|_f_ curves shown in the inset. Clearly, the carrier fluid is not frozen at these temperatures, so both the Néel and the Brown relaxation are active. Consequently, we observe *effective* relaxation times τ_eff_(T_p2_) that, in principle, can include contributions from both the above-mentioned mechanisms. Interestingly, however, we found evidence of a non-Debye behavior of the Neel and Brown relaxation times that does not follow the Rosensweig formula (Equation (3)). First, the value of the observed effective relaxation time at T = 225 K, τ_eff_ is 5 × 10^−5^ s, is three orders of magnitude more that than its Néel counterpart τ_N_~8 × 10^−8^ s (estimated by extrapolating the DBF analysis). Therefore, $\frac{1}{\tau_{N}}\gg \frac{1}{\tau_{eff}}$, which renders Equation (3) unphysical, regardless of the value of the Brown relaxation time τ_B_. As a result, we decided to make an attempt at fitting the τ_eff_(T_p2_) data starting with a model based on the hydrodynamic Brown relaxation, $\tau_{B}(T)=\frac{3\eta \left(T\right)V_{H}}{k_{B}T}$, (Equation (2)), where V_H_ is the average hydrodynamic volume of the nanoparticles and η(T) is the temperature-dependent viscosity of the carrier fluid. η varies with T according to an activation law $\eta \left(T\right)=\eta_{0}\exp\left[E^{\prime }/k_{B}(T-{T_{0}}^{\prime }\right]$ where E′ is the activation energy and T_0_′ is the viscosity divergence temperature [41]. Combining this with Equation (2) yields the following:

(4) $$\tau_{B}^{-1}\left(T\right)=\frac{T}{\gamma_{0}}\exp\left[-\frac{E^{\prime }}{k_{B}\;\left(T-{T_{0}}^{\prime }\right)}\right]$$

with $\gamma_{0}=\frac{3\eta V_{H}}{k_{B}}$. The dashed line in Figure 5 is the best fit of Equation (4) to the τ_eff_(T_p2_) data. The calculation converges with low residuals (χ^2^ = 2.54) upon the variation of three independent parameters and yields $\gamma_{0}=\frac{3\eta_{0}V_{H}}{k_{B}}$ = 1.6 × 10^−5^ s·K, E′/k_B_ = 312 K, and T_0_′ = 178 K. The quality of the fit confirms that the temperature dependence of the observed relaxation time at T_p2_ is excellently described by a model that only includes contributions from the Brown relaxation. This indicates that the physical rotation of the nanoparticles in the fluid—either individually or as aggregates—is entirely responsible for the superspin relaxation at these temperatures. 

It is also important to mention that the Néel mechanism is still active at T_p2_, and the rapid superspin flips within the nanoparticle might affect the Brown kinetics. Yet, there is no coupling between the Néel and the Brown relaxation according to Equation (3), i.e., within the framework of the Debye model. This is further demonstrated by the observation that the high-temperature peaks vanish in the highly diluted samples, whereas the low-temperature peaks (which depend exclusively on the Néel relaxation) are not affected. This is significant because is shows that weakening the interparticle interactions via dilution, which inhibits the Brownian relaxation around 225 K (most likely by preventing nanoparticle aggregation, which changes V_H_) also inhibits the overall relaxation, which would clearly not happen if the Neel–Brown coupling was governed by the Rosensweig formalism.

Our results are important because they provide experimental evidence to support the hypothesis that non-Debye relaxation occurs in interacting ferrofluids. This might lead to new approaches to design highly effective ferrofluids for magnetic hyperthermia therapy.

## 4. Summary

We carried out ac-susceptibility measurements on Fe_3_O_4_/Isopar M ferrofluids of different concentrations/dilutions to investigate the effect of the interparticle interactions on the collective superspin dynamics of SPN ensembles. Our temperature-resolved data collected at different frequencies, χ″ vs. T|_f_, show that the interplay between the Néel and the Brown relaxation mechanisms strongly depends on the temperature and the strength of the interparticle interactions. At low temperatures (around 75 K), where the carrier fluid is frozen and the Néel relaxation is the only active mechanism, we found that the observed temperature dependence of the relaxation time is well described by the Dorman–Bessais–Fiorani (DBF) model, $\tau_{N}\left(T\right)=\tau_{r}\exp\left(\frac{E_{B}+E_{ad}}{k_{B}T}\right)$, for all the ferrofluid dilutions studied here (δ = 1, δ = 0.2, δ = 0.02, δ = 0.013, and δ = 0.01). The best fit to the $\tau_{N}\left(T\right)$ data from the most diluted sample, δ = 0.01, yields τ_r_ = 3.4 × 10^−9^ s, and (E_B_ + E_ad_)/k_B_ = 900 K. At higher temperatures (around 225 K), both the Néel and the Brown relaxation are active, and the observed relaxation times, τ_eff_, may include contributions from both mechanisms. Yet, we found evidence that the Debye model and Rosensweig formula, $\frac{1}{\tau_{eff}}=\frac{1}{\tau_{N}}+\frac{1}{\tau_{B}}$, do not describe the magnetization dynamics observed in the presence of strong interparticle interactions in the densest sample, δ = 1. First, τ_eff_ is 5 × 10^−5^ s at 225 K, three orders of magnitude more than its Néel counterpart, τ_N_~8 × 10^−8^ s, so $\frac{1}{\tau_{N}}\gg \frac{1}{\tau_{eff}}$, which is obviously at odds with the Rosensweig formula. In addition, the observed temperature dependence of the effective relaxation time is described by $\tau_{eff}^{-1}\left(T\right)=\tau_{B}^{-1}\left(T\right)=\frac{T}{\gamma_{0}}\exp\left[-\frac{E^{\prime }}{k_{B}\left(T-{T_{0}}^{\prime }\right)}\right]$, a model that only accounts for the hydrodynamic Brown relaxation. The best fit yields $\gamma_{0}=\frac{3\eta V_{H}}{k_{B}}$ = 1.6 × 10^−5^ s·K, E′/k_B_ = 312 K, and T_0_′ = 178 K. Moreover, the higher-temperature peaks of χ″ are only present in the dense samples (δ = 1 and δ = 0.2), indicating that the formation of larger hydrodynamic particles via aggregation, which is responsible for observed Brownian relaxation, is inhibited by dilution.

## Data Availability

The data presented in this study are available on reasonable request from the corresponding author.
